# The clinical and genetic features in patients coexisting primary breast and thyroid cancers

**DOI:** 10.3389/fendo.2023.1136120

**Published:** 2023-05-09

**Authors:** Jingyao Fu, Miao He, Qiong Wu, Xiangkai Zhang, Xin Qi, Keyu Shen, Xiaochun Wang, Guang Zhang

**Affiliations:** ^1^ Department of Thyroid Surgery, China-Japan Union Hospital of Jilin University, Changchun, Jilin, China; ^2^ Department of Oral-Maxillofacial-Thyroid Oncosurgery, Jilin Cancer Hospital, Changchun, Jilin, China; ^3^ Department of Anesthesia, The Second Hospital of Jilin University, Changchun, Jilin, China; ^4^ Department of Breast Surgery, China-Japan Union Hospital of Jilin University, Changchun, Jilin, China; ^5^ Department of Thyroid and Breast Surgery, Jining No.1 People’s Hospital, Jining, Shandong, China

**Keywords:** breast cancer, thyroid cancer, COMP, prognosis, standardized incidence ratio (SIR)

## Abstract

**Background:**

We attempted to examine the clinical characteristics in patients with breast cancer (BC) and thyroid cancer (TC); explore the potential mechanisms of tumorigenesis and progression.

**Methods:**

Using the Surveillance, Epidemiology, and End Result Program-9 (SEER-9) database, a retrospective study (1975-2017) was conducted on patients with BC and TC. We identified the common differentially expressed genes involved in BC and TC using the Gene Expression Omnibus database (GEO). Immunohistochemical staining (IHC) was performed to verify the expression of the hit gene in patients with co-occurrence of BC and TC. Using The Cancer Genome Atlas (TCGA) database, the relationship between gene expression and clinicopathological characters was determined. Gene set enrichment analysis (GSEA) was used to identify the pathways enriched in BC and TC.

**Results:**

BC patients had a higher predisposition to develop TC (standardized incidence ratio, SIR: 1.29) and vice-versa (SIR: 1.12). Most of these patients were differentiated thyroid carcinoma (DTC) and hormone receptor (HR) - positive BC. The mRNA expression of COMP (Cartilage oligomeric matrix protein) was significantly overexpressed in BC and TC by analyzing the GEO database. The protein expression of COMP was increased in both BC and TC tissues obtained from the same patients validated by IHC. COMP was correlated with worse OS in BC (stage II-IV) and TC; it was the independent factor for prognosis of BC. GSEA indicated that the estrogen response and epithelial-mesenchymal transition (EMT) pathways were significantly enriched in both TC- and BC- COMP overexpressed groups.

**Conclusion:**

The co-occurrence risk of BC and TC in the same individual is higher than in the general population. Overexpression of COMP could promote oncogenesis and progression in patients with BC and TC through estrogen signaling and EMT pathways.

## Introduction

Globally, breast cancer (BC) is the most commonly diagnosed malignancy in females, accounting for about 25.1% of all cancer diagnoses (1). It is reported that BC survivors have an increased risk of developing a second primary tumor, in particular, thyroid cancer (TC) (2, 3). Although the global incidence of TC is on the rise in recent decades, the patients have relatively higher survival rates. However, survivors of TC have a higher risk of developing BC than the general population. The risk of BC in TC survivors increased from 21% to 89%, while that of TC in BC survivors increased from 31% to 73% (4).

Previous studies have reported co-incidences of BC and TC occurring either synchronously or as metachronous malignancies. A retrospective case-controlled study by An et al. (4) reports that in the five-year follow up of 4243 TC patients, 55 developed BC; the standardized incidence ratio (SIR) was 2.45. Conversely, in 6.2-year follow up of 6833 BC patients, 88 had subsequently developed TC; SIR of 2.18. The expression of estrogen and progesterone receptors (ER and PR) in the tissues of BC patients with TC was significantly higher than those of patients with BC only (5). In the case of TC co-occurring with BC, smaller tumor and higher follicular thyroid cancer (FTC) is reported in comparison with TC-only patients (6).

Taken together, these studies suggest that BC and TC may have some common etiologies, such as genetic susceptibility, hormonal and environmental factors. Previous studies have reported the association between thyroid hormones and breast cancer risk; abnormally high free-thyroxine is positively correlated while higher thyroid-stimulating hormone level in the euthyroid range is negatively correlated with breast cancer (7–9). Higher estrogen level and obesity are also implicated in the development of TC and BC (10). Studies also reported that there is neither any significant increase in BC incidence after radioactive iodine treatment nor any significant increase in the risk of TC after radiotherapy for BC (11, 12). As for the genetic correlation, previous studies report that the mutations in the tumor suppressor PTEN are correlated with Cowden syndrome, and significantly increases the risk of breast and thyroid cancers (13). In addition, PARP4 germline mutations are implicated as potential susceptibility factors in synchronous thyroid and breast cancers (14). Common pathogenic factors for BC and TC are not clearly understood. In the present study, we investigate the association between the two cancers and the underlying genetic factors for tumorigenesis and progression of the malignancies. We found that the risk of subsequently developing BC or TC in TC or BC patients, respectively, was higher than in general population. COMP (Cartilage oligomeric matrix protein) was significantly overexpressed in BC and TC, which was an independent factor for the prognosis of BC. COMP overexpression had significantly enriched the estrogen response and EMT pathways. These results of this study may have implications in clinical diagnosis, treatment, and prognosis of the associated tumors.

## Materials and methods

### Data collection and analyses

Data collection was done from the SEER-9 database (https://seer.cancer.gov/) using the SEER^*^Stat software. The database includes approximately 10% population of the United States. To analyze the association between TC and BC, we identified patients according to primary tumor site codes; C73.9 for TC and C50.0-C50.9 for BC. From 1975 to 2017, a total of 1982 TC patients had subsequently developed BC and 2098 BC patients had developed TC. Tumor histology, incidence rate, and survival information of the double primary malignancies were collected and analyzed.

SIR was calculated using the multiple primary-SIR program of SEER*Stat and used to compare the incidence risk of BC or TC in previously diagnosed BC or TC patients with that in normal population. SIR = standardized incidence rate of BC (TC) patients to develop TC (BC)/standardized incidence rate of the general population to develop TC (BC). SIR > 1 and *p-*value < 0.05 suggested that patients with BC or TC have a significantly higher risk of developing secondary tumors than the normal population.

Patients with coexisting breast and thyroid cancers were grouped by primary cancer, the histological subtype of TC, and the hormonal status of BC for survival analysis. Survival curves were plotted using the Kaplan-Meier method. Data analysis was performed using SPSS v25 (SPSS Inc. Chicago, Illinois, USA), and *p* < 0.05 was considered statistically significant.

### Identification of differentially expressed genes

Two gene expression datasets (GSE3467 (15) and GSE61304 (16)) were downloaded from the Gene Expression Omnibus (GEO) database (https://www.ncbi.nlm.nih.gov/geo/). Paired, 9-papillary thyroid carcinoma (PTC) and 9-normal tissue samples were obtained from GSE3467. Sixty-two-breast tumor samples and 4-normal breast epithelium samples were obtained from GSE61304. The platform used for both datasets was GPL570 [HG-U133_Plus_2] (17) Affymetrix Human Genome U133 Plus 2.0 Array.

NetworkAnalyst (https://www.networkanalyst.ca/) was used to identify the differentially expressed genes (DEGs) in the breast-, thyroid-cancer, and normal tissue samples (*P*-value <0.05; |logFC| ≥1). Volcano plots were generated in R v4.0.3 using the ggplot2 package. Common DEGs from two datasets were prioritized using Draw Venn Diagram (http://bioinformatics.psb.ugent.be/webtools/Venn/).

### COMP expression in The Cancer Genome Atlas (TCGA) patients

COMP mRNA-seq data for THCA and BRCA were downloaded from the TCGA database (https://portal.gdc.cancer.gov/). The package edgeR in R v4.0.3 was used to normalize the raw RNA counts and identify the difference in COMP expression between the normal and tumor tissues. Further, diagnostic capability of COMP in TC and BC was calculated using ROC curves.

### Association of COMP overexpression with clinical characteristics of TC and BC patients

Clinical data for TC and BC patients were obtained from the TCGA database. A total of 501 TC patients and 1090 BC patients had both the mRNA data and complete clinic information. The clinicopathological characteristics included TNM stage, tumor size, lymph node metastasis, distant metastasis, ER status, PR status, human epidermal growth factor receptor 2 (HER2) status, age, and gender. According to the optimal threshold of COMP in BC and TC (51.85 and 17.81 FPKM, respectively), patients were classified into two groups (high/low). The correlation between COMP expression and clinical characteristics was calculated by Chi-square test.

### Survival analysis

Kaplan-Meier (K-M) analysis was used to compare the overall survival (OS) between high and low expression COMP groups. Univariate and multivariate COX analyses were also performed to identify the independent factors related to OS.

### Gene set enrichment analysis

GSEA was performed using TCGA (THCA and BRCA) datasets to identify the potential enriched pathways which were correlated with COMP overexpression. Pathways with *p-*value < 0.05 and the false discovery rate (FDR) < 0.25 and | Normalized Enrichment Score (NES)| ≥1 were considered significantly enriched.

### Correlated genes of COMP

Based on the TCGA database, The correlation of genes in the enriched pathways with COMP in TC and BC was evaluated by Spearman correlation analysis. Further, the expression and the correlation of these genes with OS in TC and BC were examined.

### Immunohistochemical staining of patients with BC and TC

The paraffin-embedded BC, TC, and paired normal tissues of 7-double malignancies from patients coexisting with BC and TC who underwent surgeries were collected. Tissue sections were deparaffinized and antigen retrieval was done using citrate buffer. The sections were incubated overnight at 4°C with a rabbit polyclonal antibody against COMP (1:400; Abcam, Cambridge, UK). Diaminobenzidine was added to the sections and incubated at room temperature. Counterstaining was done with hematoxylin. Image-Pro Plus 6.0 was used to quantitatively analyze IHC images. The COMP level was represented by the average optical density (AOD). AOD = integrated optical density (IOD)/area.

### Statistical analysis

R v4.0.3 and SPSS v25 (SPSS Inc. Chicago, Illinois, USA) were used for statistical analysis. Count data were expressed as rate (%), and measurement data were expressed as mean ± standard deviation. Chi-square analysis and Fisher’s exact test were used to compare categorical variables. The Wilcoxon and Kruskal-Wallis tests are used to compare continuous variables. The ROC curve was used to calculate the diagnostic capability of COMP for TC and BC. The survival curve was plotted using the Kaplan-Meier method. Univariate and multivariate COX regression were used to analyze the clinical features affecting the survival of BC and TC patients. Spearman correlation analysis was used to evaluate the correlation between genes in the COMP enrichment pathway and COMP in TC and BC. Image-Pro Plus 6.0 was used for the quantitative analysis of IHC images. COMP levels are expressed as average optical density (AOD). IHC results are plotted by GraphPad Prism 9. P <0.05 was the threshold of significance.

## Results

### Patients with breast cancer and thyroid carcinoma

From 1975 to 2017, 2098 out of 87,1359 BC patients subsequently developed TC (BC-1^st^); median time between diagnosis of BC and subsequent diagnosis of TC was 69 months. During the same period, 1982 out of 97,530 TC patients subsequently developed BC (TC-1^st^). There were 72,896 female TC patients, of which 1975 were subsequently diagnosed with BC. Seven of 2463 male TC patients were diagnosed with BC. The median between the diagnoses was 111 months. Among BC-1^st^ patients (**Table 1**), 55.1% BC patients were HR-positive and 91.3% of them subsequently developed DTC. In 73.5% TC-1^st^ patients (**Table 2**), the subsequent BC subtype was HR-positive.

**Table 1:** Patients with BC subsequently diagnosed as TC.

BC	BC-1^st^					Total
	TC					
	PTC	FTC	MTC	ATC	Others	
HR Positive	1005(47.9%)	57(2.7%)	19(0.9%)	21(1.0%)	54(2.6%)	1156(55.1%)
HR Negative	185(8.8%)	16(0.8%)	4(0.2%)	2(0.1%)	8(0.4%)	215(10.2%)
Unknown	587(28.0%)	66(3.1%)	11(0.5%)	4(0.2%)	59(2.8%)	727(34.7%)
Total	1777(84.7%)	139(6.6%)	34(1.6%)	27(1.3%)	121(5.8%)	2098(100.0%)

PTC, papillary thyroid carcinoma; FTC, follicular thyroid carcinoma; MTC, medullary thyroid carcinoma; ATC, anaplastic thyroid carcinoma; HR, hormone receptor.

**Table 2:** Patients with TC subsequently diagnosed as BC.

TC	TC-1st			Total
	BC			
	HR Positive	HR Negative	Unknown	
PTC	1218(61.5%)	197(9.9%)	234(11.8%)	1649(83.2%)
FTC	153(7.7%)	20(1.0%)	37(1.9%)	210(10.6%)
MTC	24(1.2%)	7(0.4%)	7(0.4%)	38(1.9%)
ATC	3(0.2%)	0(0.0%)	0(0.0%)	3(0.2%)
Others	59(3.0%)	7(0.4%)	16(0.8%)	82(4.1%)
Total	1457(73.5%)	231(11.7%)	294(14.8%)	1982(100.0%)

PTC, papillary thyroid carcinoma; FTC, follicular thyroid carcinoma; MTC, medullary thyroid carcinoma; ATC, anaplastic thyroid carcinoma; HR, hormone receptor.

### SIR of BC-1^st^ and TC-1^st^

SIR was used to examine the theoretical linkages in the etiology of the two cancers, by comparison of subsequent cancer experiences with the number of cancers that would be expected based on incidence rates in the general population. SIR>1 and P<0.05 indicate that BC or TC survivors have a higher risk of developing another primary cancer than the general population. For BC-1^st^ group (**Table 3**), the SIR was 1.29 (95% CI: 1.23-1.35) for both females and males. Male BC patients had higher SIR of 3.0 (95% CI: 1.50-5.37), while that of the females was 1.28 (95% CI: 1.22-1.35). On comparing the hormone receptor (HR) status, HR-positive BC had a higher risk of subsequent TC development as compared to HR-negative BC (1.37 and 1.18, respectively). For the TC-1^st^ group (**Table 4**), the SIR was 1.12 (95% CI: 1.07-1.17) for both females and males. However, only the female TC patients had a significant SIR of 1.12 (95% CI: 1.07-1.17). Additionally, DTC patients had a higher risk of developing BC. Among them, the SIR of FTC patients was 1.23; the excess risk was 4.51, which was higher than that in PTC patients (1.11). Our results also indicated that in the advanced BC stages, SIR was higher for subsequent TC; in TC, stage III patients had significantly higher risk of developing BC.

**Table 3:** SIR of Patients with TC after BC.

Variable	SIR	CI Lower	CI upper	Excess Risk
Total	1.29**	1.23	1.35	0.60
Sex				
Male	3.00**	1.50	5.37	2.24
Female	1.28**	1.22	1.35	0.59
Age at diagnosis				
0-49years	1.24**	1.15	1.35	0.62
50+years	1.31**	1.24	1.39	0.60
HR status				
HR Positive	1.37**	1.29	1.46	0.94
HR Negative	1.18*	1.02	1.36	0.46
Stage				
Stage I	1.41**	1.25	1.59	1.24
Stage II	1.73**	1.52	1.97	2.26
Stage III	2.31**	1.87	2.83	4.11
Stage IV	1.77	0.99	2.92	2.33

*p<0.05; **p<0.01; SIR, standardized incidence ratio; HR, hormone receptor.

**Table 4:** SIR of Patients with BC after TC.

Variable	SIR	CI Lower	CI upper	Excess Risk
Total	1.12**	1.07	1.17	2.27
Sex				
Male	1.42	0.61	2.81	0.1
Female	1.12**	1.07	1.17	2.9
Age at diagnosis				
0-49years	1.12**	1.05	1.19	1.7
50+years	1.12**	1.06	1.2	3.47
Histological type				
PTC	1.11**	1.06	1.17	2.11
FTC	1.23**	1.08	1.41	4.51
MTC	1.15	0.84	1.54	2.7
ATC	1.98	0.54	5.08	19.98
Stage				
Stage I	1.10	0.99	1.23	1.68
Stage II	0.94	0.69	1.25	-1.39
Stage III	1.28*	1.03	1.58	6.18
Stage IV	1.06	0.72	1.52	1.21

*p<0.05; **p<0.01; SIR, standardized incidence ratio; PTC, papillary thyroid carcinoma; FTC, follicular thyroid carcinoma; MTC, medullary thyroid carcinoma; ATC, anaplastic thyroid carcinoma.

### Survival analysis in BC-1^st^ and TC-1^st^ groups

**Figure 1:**
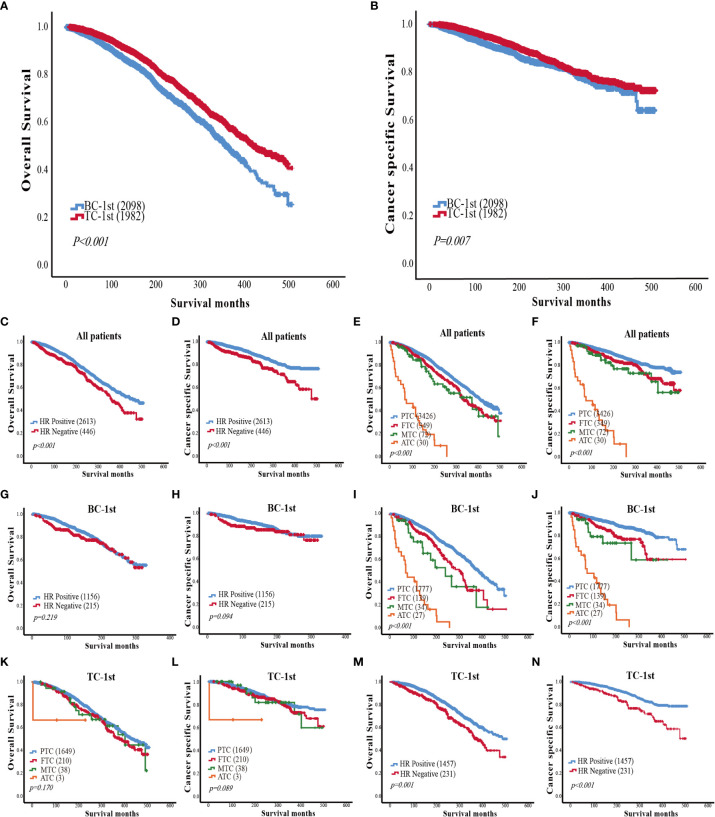
**(A)** Comparison of overall survival curves between BC-1st and TC-1st patients. **(B)** Comparison of cancer-specific survival curves between BC-1st and TC-1st patients. **(C)** Overall survival curves based on the HR status of patients with BC and TC. **(D)** Cancer-specific survival curves based on the HR status of patients with BC and TC. **(E)** Overall survival curves based on TC histological subtype of patients with BC and TC. **(F)** Cancer-specific survival curves based on TC histological subtype of patients with BC and TC. **(G)** Overall survival curves based on the HR status of BC-1st patients. **(H)** Cancer-specific survival curves based on the HR status of BC-1st patients. **(I)** Overall survival curves based on TC histological subtype of BC-1st patients. **(J)** Cancer-specific survival curves based on TC histological subtype of BC-1st patients. **(K)** Overall survival curves based on TC histological subtype of TC-1st patients. **(L)** Cancer-specific survival curves based on TC histological subtype of TC-1st patients. **(M)** Overall survival curves based on the HR status of TC-1st patients. **(N)** Cancer-specific survival curves based on the HR status of TC-1st patients.

Kaplan-Meier analyses were used to analyze the survival of patients in the BC-1^st^ and TC-1^st^ groups. Either the overall survival curves (**Figure 1A**) or cancer-specific (Death due to TC or BC) (**Figure 1B**) survival curves for BC-1^st^ patients declined faster than that for TC-1^st^ patients. The three-year survival rates of BC-1^st^ and TC-1^st^ patients were 97.6% and 98.9%, the five-year survival rates were 95.7% and 97.8%, and the ten-year survival rates were 88.2% and 92.5%, respectively.

Then we analyzed the impact of HR status and TC histological subtype on the survival of patients with BC and TC. The overall and cancer-specific survival curves of HR-negative patients declined significantly faster as compared to HR-positive patients (*p*<0.001) (**Figures 1C, D**). In the TC histological subtype, patients with PTC had the best survival, while patients with anaplastic thyroid carcinoma (ATC) had the worst survival. The survival of FTC patients was worse than that of PTC patients but better than the medullary thyroid carcinoma (MTC) patients (*p*<0.001) (**Figures 1E, F**).

In the BC-1st patients, the effects of HR status on overall survival and cancer-specific survival were not statistically significant (**Figures 1G, H**). However, PTC was correlated with a better OS in BC-1^st^ patients (**Figures 1I, J**). In the TC-1^st^ patients, there was no significant difference in survival among different histological subtypes of TC (**Figures 1K, L**). However, in the TC-1^st^ patients, HR-positive BC patients had a better OS (*p*<0.001) (**Figures 1M, N**).

### COMP was upregulated in BC and TC tissues compared to normal tissues

**Figure 2:**
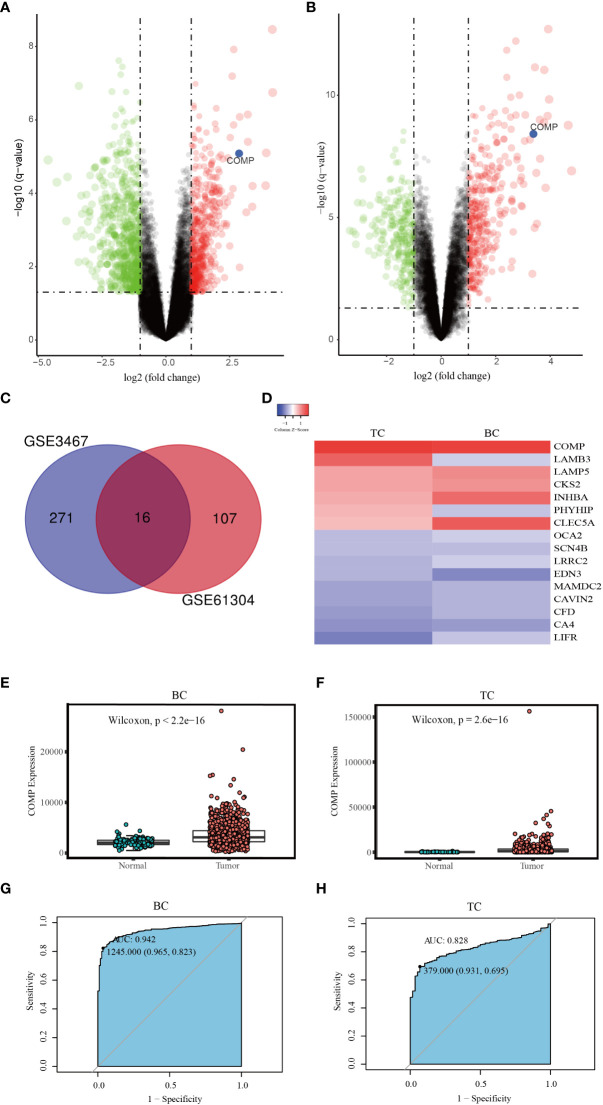
**(A)** Volcano map of DEGs between BC and normal tissues (GSE61304). **(B)** Volcano map of DEGs between TC and normal tissues (GSE3467). **(C)** The Venn diagram of all DEGs in the two datasets; **(D)** The expression level of DEGs in two datasets. **(E)** The differential expression of COMP between BC and normal tissues. **(F)** The differential expression of COMP between TC and normal tissues. **(G)** ROC curve of COMP upregulation for BC. **(H)** ROC curve of COMP upregulation for TC.

To explore the common genetic mechanism of BC and TC, differential expression analysis was performed. Using GSE61304, a total of 123 DEGs were identified; 29 genes were up-regulated while 94 were down-regulated in BC (**Figure 2A**). In the GSE3467 dataset, a total of 287 DEGs were identified; 161 genes were up-regulated while 126 were down-regulated in TC (**Figure 2B**). Common DEGs in BC and TC are depicted using a Venn diagram as shown in **Figure 2C**. A total of 16 common DEGs were obtained, including 5 genes which were up-regulated (COMP, CKS2, CLEC5A, LAMP5, INHBA) and 9 genes which were down-regulated (LIFR, LRRC2, CAVIN2, OCA2, CA4, SCN4B, EDN3, MAMDC2, CFD). Two DEGs were up-regulated in TC but down-regulated in BC (PHYHIP, LAMB3). A heat map was generated to represent the expression of these DEGs in the two datasets as shown in **Figure 2D**. Noticeable, COMP is significantly highly expressed in both BC and TC.

Then using data from the TCGA database, we further confirmed that the expression of COMP was significantly upregulated in BC as well as in the TC patients as compared to the normal tissues (**Figures 2E, F**). Thus, the diagnostic value of COMP for BC and TC patients was evaluated using ROC curves. The AUC of COMP for BC and TC was 0.942 and 0.828, respectively (**Figures 2G, H**).

### Association between COMP expression and clinical characteristics

**Figure 3:**
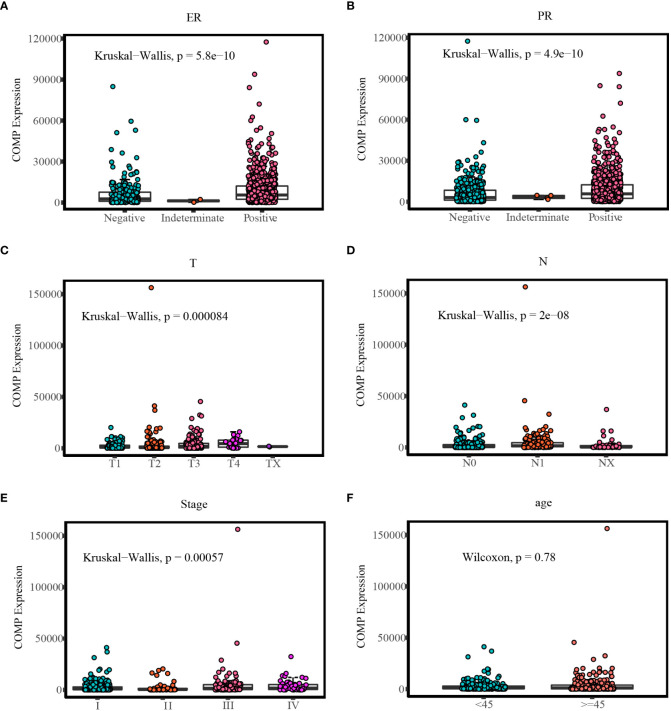
Comparison of COMP expression in different clinic groups: **(A)** Different COMP expression levels in ER status of BC; **(B)** Different COMP expression levels in PR status of BC; **(C)** Different COMP expression levels in T stages of TC; **(D)** Different COMP expression levels in N stages of TC; **(E)** Different COMP expression levels in TNM stages of TC; **(F)** Different COMP expression levels in age groups of TC.

In BC patients, there was a significant correlation between the expression of COMP and ER or PR status (**Table 5**). As shown in **Table 6** COMP was significantly correlated with the TNM stage, tumor size, lymph nodes metastasis, and age of TC patients. Furthermore, the association between COMP expression and clinic characteristics of BC or TC patients was verified using the COMP mRNA expression data as a continuous variable (**Figure 3**).

**Table 5:** Association between COMP expression and clinic phenotype of BC patients.

	COMP		p
	Low	High	
Gender			1.0
Female	757(98.8%)	321(99.1%)	
Male	9(1.2%)	3(0.9%)	
Age			0.607
<45	109(14.2%)	50(15.4%)	
≧45	657(85.8%)	274(84.6%)	
ER status			<0.001
Negative	189(24.7%)	48(14.8%)	
Indeterminate	2(0.3%)	0(0.0)	
Positive	537(70.1%)	266(82.1%)	
NA	38(5.0%)	10(3.1%)	
PR status			0.001
Negative	264(34.5%)	79(24.4%)	
Indeterminate	4(0.5%)	0(0.0)	
Positive	459(59.9%)	235(72.5%)	
NA	39(5.1%)	10(3.1%)	
Her2 status			0.079
Negative	530(69.2%)	232(71.6%)	
Equivocal	24(3.1%)	7(2.2%)	
Positive	120(15.7%)	62(19.1%)	
NA	92(12.0%)	23(7.1%)	
Tumor size			0.435
T1	186(24.3%)	93(28.7%)	
T2	449(58.6%)	182(56.2%)	
T3	101(13.2%)	36(11.1%)	
T4	27(3.5%)	13(4.0%)	
TX	3(0.4%)	0(0.0%)	
Lymph nodes metastasis			0.847
N0	368(48.0%)	146(45.1%)	
N1	246(32.1%)	114(35.2%)	
N2	83(10.8%)	37(11.4%)	
N3	54(7.0%)	22(6.8%)	
NX	15(2.0%)	6(1.5%)	
Distant metastasis			0.509
M0	643(83.9%)	264(81.5%)	
M1	16(2.1%)	6(1.9%)	
MX	107(14.0%)	54(16.7%)	
Stage			0.618
Stage I	124(16.2%)	57(17.6%)	
Stage II	438(57.2%)	181(55.9%)	
Stage III	177(23.1%)	70(21.6%)	
Stage IV	14(1.8%)	6(1.9%)	
Stage X	13(1.7%)	10(3.1%)	

ER, estrogen receptor; PR, progesterone receptor; Her2, human epidermal growth factor receptor 2.

**Table 6:** Association between COMP expression and clinic phenotype of TC patients.

	COMP		p
	Low	High	
Gender			0.479
Female	277(73.9%)	89(70.0%)	
Male	98(26.1%)	37(29.4%)	
Age			0.042
<45	196(52.3%)	79(62.7%)	
≧45	179(47.7%)	47(37.3%)	
Tumor size			<0.001
T1	118(31.5%)	25(19.8%)	
T2	134(35.7%)	30(23.8%)	
T3	110(29.3%)	59(46.8%)	
T4	11(2.9%)	12(9.5%)	
TX	2(0.5%)	0(0.0%)	
Lymph nodes metastasis			<0.005
N0	180(48.0%)	48(38.1%)	
N1	152(40.5%)	71(56.3%)	
NX	43(11.5%)	7(5.6%)	
Distant metastasis			0.102
M0	201(53.6%)	81(64.3%)	
M1	7(1.9%)	2(1.6%)	
MX	167(44.5%)	43(34.1%)	
Stage			0.001
Stage I	226(60.3%)	55(43.7%)	
Stage II	42(11.2%)	10(7.9%)	
Stage III	69(18.4%)	42(33.3%)	
Stage IV	36(9.6%)	19(15.1%)	
Stage X	2(0.5%)	0(0.0%)	

### Survival analysis of COMP

**Figure 4:**
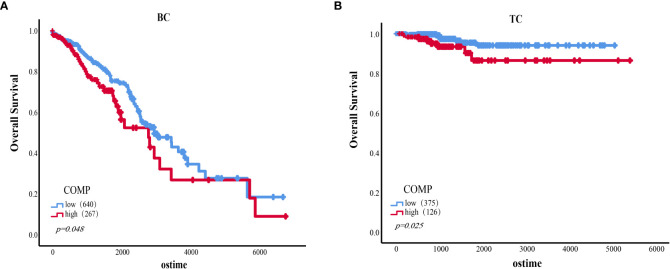
**(A)** The association of the expression level of COMP with OS of BC patients (Stage II-IV). **(B)** The association of the expression level of COMP with OS of TC patients.

Survival analysis based on TCGA data indicated that the expression of COMP was correlated with OS of patients in stage II-IV BC (*p*=0.048) (**Figure 4A**). Overexpression of COMP was significantly associated with poor OS of TC patients (*p*=0.025) (**Figure 4B**). In addition, Cox regression analysis showed that COMP levels, age, PR status, and lymph nodes metastasis were independent factors of poor OS in BC patients (**Table 7**). In the TC patients, univariate analysis showed that COMP, age, tumor size, distance metastasis, and TNM stage were correlated with OS. However, in multivariate analysis, COMP was not an independent factor of OS (**Table 8**).

**Table 7:** Univariate and multivariate Cox regression analyses in BC patients.

Variables	Univariable analysis		Multivariable analysis	
	HR	*P* value	HR	*P* value
COMP	1.437(1.001-2.063)	0.049	1.667(1.152-2.413)	0.007
Gender	1.415(0.197-10.168)	0.730	–	–
Age	1.682(1.023-2.766)	0.041	1.845(1.121-3.039)	0.016
ER status	1.717(1.267-2.326)	<0.001		
PR status	1.806(1.337-2.439)	<0.001	1.968(1.462-2.649)	<0.001
Her2 status	1.040(0.853-1.268)	0.698	–	–
Tumor size	1.237(0.994-1.539)	0.056	–	–
Lymph nodes metastasis	1.346(1.119-1.621)	0.002	1.375(1.143-1.655)	0.001
Distant metastasis	1.821(1.142-2.904)	0.012	–	–

HR, hazard ratio; ER, estrogen receptor; PR, progesterone receptor; Her2, human epidermal growth factor receptor 2.

**Table 8:** Univariate and multivariate Cox regression analyses in BC patients.

Variables	Univariable analysis		Multivariable analysis	
	HR	*P* value	HR	*P* value
COMP	2.917(1.095-7.776)	0.032	–	–
Gender	1.924(0.696-5.319)	0.207	–	–
Age	76.802(1.339-4404.984)	0.036	–	–
Tumor size	2.599(1.411-4.789)	0.002	–	–
Lymphnodes metastasis	0.912(0.443-1.877)	0.803	–	–
Distant metastasis	2.132(0.854-5.322)	0.105	–	–
Stage	2.421(1.540-3.805)	<0.001	–	–

### Gene set enrichment analysis

**Figure 5:**
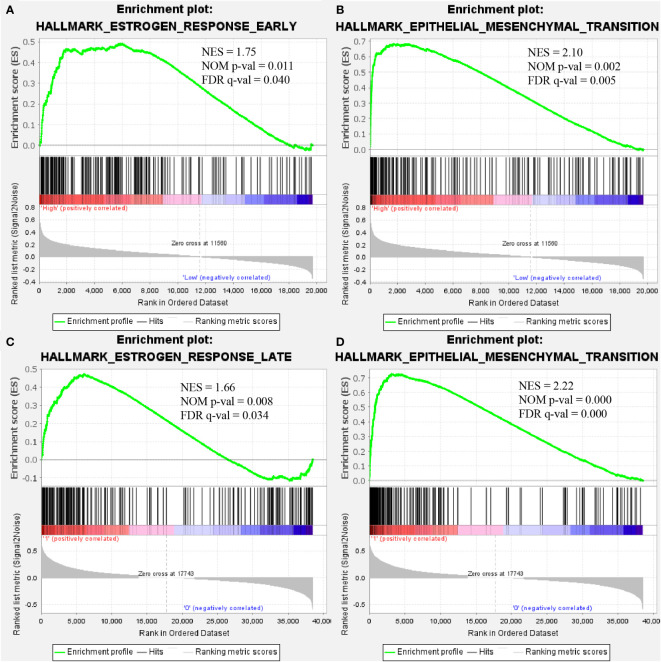
COMP overexpression was significantly associated with “estrogen response early” **(A)** and “epithelial-mesenchymal transition” **(B)** in BC. COMP overexpression was also associated with “estrogen response late” **(C)** and “epithelial-mesenchymal transition” **(D)** in TC.

GSEA was performed to identify the enriched pathways associated with upregulated COMP in TC and BC (**Figure 5**). Results indicated that the estrogen response early and estrogen response late pathways were enriched in BC and TC, respectively; the epithelial-mesenchymal transition (EMT) pathways were significantly enriched in both BC and TC.

### COMP-related genes in estrogen response pathway and EMT pathway

**Figure 6:**
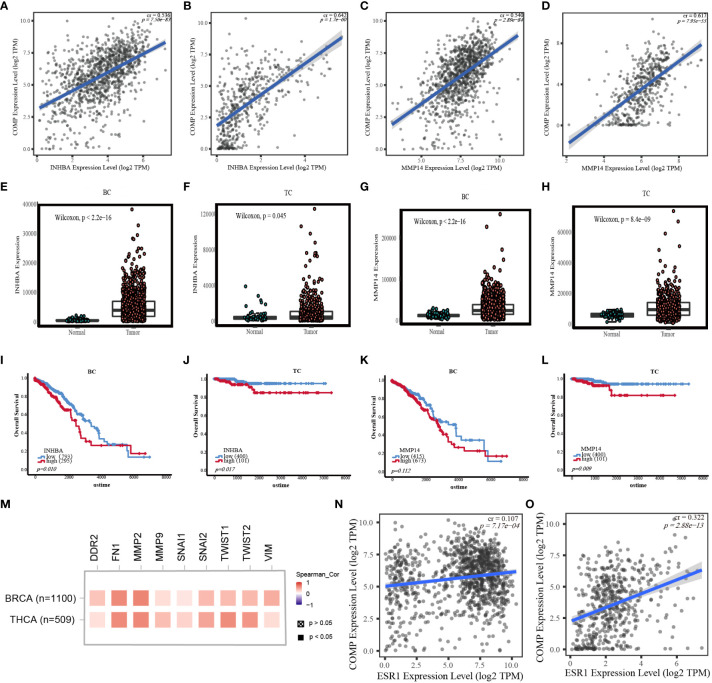
**(A)** Correlation between INHBA expression and COMP mRNA expression in BC. **(B)** Correlation between INHBA expression and COMP mRNA expression in TC. **(C)** Correlation between MMP14 expression and COMP mRNA expression in BC. **(D)** Correlation between MMP14 expression and COMP mRNA expression in TC. **(E)** Differential expression of INHBA between BC and normal tissues. **(F)** Differential expression of INHBA between TC and normal tissues. **(G)** Differential expression of MMP14 between BC and normal tissues. **(H)** Differential expression of MMP14 between TC and normal tissues. **(I)** Correlation between the expression level of INHBA and OS in BC patients. **(J)** Correlation between the expression level of INHBA and OS in TC patients. **(K)** Correlation between the expression level of MMP14 and OS in BC patients. **(L)** Correlation between the expression level of MMP14 and OS in TC patients. **(M)** Correlation between COMP expression and EMT marks expression. **(N)** Correlation between ESR1 expression and COMP mRNA expression in BC. **(O)** Correlation between ESR1 expression and COMP mRNA expression in TC.

INHBA and MMP14 in EMT pathways were correlated with COMP expression (**Figures 6A–D**) for both BC and TC. INHBA and MMP14 were significantly overexpressed in BC and TC tissues as compared to normal tissues (**Figures 6E–H**). K-M analysis showed that MMP14 overexpression was significantly correlated with unfavorable OS in TC (*p* = 0.009; **Figure 6L**). INHBA expression and OS were significantly correlated in both BC and TC (*p* < 0.05, **Figures 6I, J**). However, the high expression of MMP14 was not significantly correlated with survival in BC (**Figure 6K**).

In addition, in breast and thyroid cancers, we also found that COMP was positively associated with other EMT markers (TWIST1, TWIST2, SNAI1, SNAI2, DDR2, MMP2, MMP9, FN1, VIM) and estrogen receptor-α (ESR1) (**Figures 6M–O**).

### Immunohistochemical staining of patients with BC and TC

**Figure 7:**
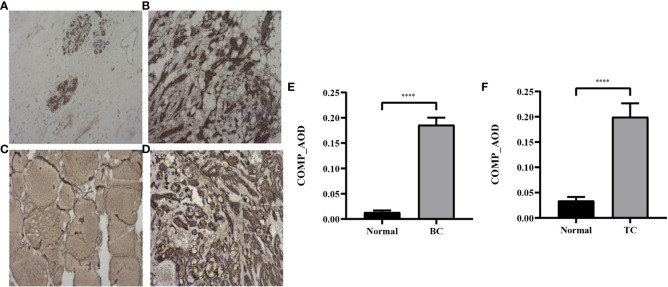
Representative IHC images of COMP in normal breast tissues **(A)**, BC tissues **(B)**, normal thyroid tissues **(C)** and TC tissues **(D)**. Magnification: ×40. **(E)** AOD of COMP in breast cancer vs normal breast tissues; **(F)** AOD of COMP in thyroid cancer vs normal thyroid tissues. ****p<0.0001.

To further validate the expression profile of COMP, immunohistochemical staining for tissues of patients with co-occurrence of BC and TC was performed. COMP was significantly overexpressed in BC and TC as compared to normal tissues, consistent with the gene expression pattern (*p*<0.0001) (**Figure 7**).

## Discussion

In this study, we showed that the risk of subsequently developing BC or TC in TC or BC patients, respectively, was higher than in general population by analyzing double primary cancer (BC and TC) data using the SEER-9 database. And the pathological or molecular subtype of patients with BC and TC was mostly DTC and HR-positive BC, respectively. Therefore, DTC and HR-positive BC may be closely related. In BC-1^st^ patients, the SIR of HR-positive BC patients was higher than that of HR-negative BC patients. DTC patients also had a significantly higher risk of developing BC. In addition, patients with higher TNM stages had a higher risk of developing another primary cancer.

We also analyzed the survival of BC-1^st^ and TC-1^st^ patients. K-M analysis indicated that the overall survival and cancer-specific survival of BC-1^st^ was poorer than that of TC-1^st^ patients. This may be attributed to the better prognosis of TC patients. Additionally, the median time for TC-1^st^ (111 months) was longer than the median time for BC-1^st^ (60 months). Further, the impact of HR status and TC subtype on the survival of double primary cancer patients were also analyzed. For all patients with BC and TC, HR status and TC histological subtype were correlated with the survival of patients. However, for BC-1^st^ patients, only the TC histological subtype was significantly associated with the survival of patients. Only the HR status was correlated with the OS of TC-1^st^ patients. Therefore, the OS of patients with BC and TC were correlated with the molecular or pathological subtype of the second primary cancer rather than the first primary cancer.

The association of TC and BC is complex; previous studies have aimed at evaluating this relationship. What more, An et al. (4) showed that the expression of ER and PR in co-occurrence of BC and TC was higher than in BC-only patients, suggesting that there is underlying molecular pathogenesis for BC and TC double primary cancers. We aimed to analyze the genetic mechanisms underlying the association of BC and TC. We obtained 16 common differentially expressed genes in BC and TC. In the preliminary analysis, other genes except COMP were not associated with the survival prognosis of BC or TC. In addition, COMP is highly expressed in both BC and TC, which may be a potential pathogenic gene, so we conducted further analysis on COMP.

We found that ROC curves suggested better diagnostic values of COMP in TC and BC. Studies (18, 19) have shown that COMP promotes the proliferation and migration of PTC and BC cells. Meanwhile, high COMP expression was found to be associated with poor prognosis in TC and BC patients in our study. The correlation between COMP and survival of stage II-IV BC patients may be due to the oncogenic role of COMP in advance stage BC. Papadakos et al. (20) show that serum COMP in metastatic BC patients was higher as compared to those in the early stages of BC and was significantly correlated with bone and liver metastasis. In this study, multivariate Cox analysis also confirmed that COMP overexpression was an independent factor of unfavorable OS in BC (stage II-IV). However, in TC, only univariate analysis showed that COMP overexpression correlated with poor OS; this probably contributes to the better prognosis of TC patients (16 of 501 deceased patients during the follow-up). Thus, COMP may be a potential biomarker for the diagnosis and prognosis of TC and BC patients. Besides we also found that stage III BC or TC survivors are more likely to develop another second primary cancer. Therefore, COMP may promote the progression of TC and BC, thereby increasing the risk of secondary primary cancer.

COMP also known as TSP5, is a member of the thrombospondin family (21). which is highly expressed in colon cancer, hepatocellular carcinoma, prostate cancer, lung cancer, and bladder cancer (22–24). COMP can enhance the ability of tumor cells to degrade ECM by upregulating the expression of MMP9, thereby, promoting EMT, which in turn promotes tumor invasion and metastasis (25). In addition, COMP promotes cell growth and proliferation by activating the MEK/ERK and PI3K/AKT signaling pathways (26). These observations are consistent with our results; COMP is overexpressed in BC patients and associated with ER and PR (19); GSEA indicated that COMP overexpression had significantly enriched the estrogen response and EMT pathways in both TC and BC and may promote TC and BC progression, thereby, increasing the risk of these double primary malignancies.

Our results also showed that INHBA and MMP14 in the EMT pathway were positively correlated with COMP overexpression. Both INHBA and MMP14 were upregulated in BC and TC as compared to the normal tissues. Additionally, INHBA overexpression was associated with unfavorable OS of BC and TC; high expression of MMP14 was associated with the poorer prognosis of TC patients. INHBA was one of 16 common DEGs which was upregulated in BC and TC. Activin A, a homodimer composed of two inhibin βA subunits is encoded by INHBA. Recent studies (27–29), indicate the importance of activin signaling in the progression of BC and TC. High expression of activin A induces EMT. MMP14 is a member of the membrane-anchored metalloproteinases (MMP) subfamily. This protein not only degrades extracellular matrix protein but also acts as an activator of other MMP zymogen including MMP2 and MMP9, playing a key role in tumor invasion (30).

EMT is important for the process of tumor metastasis. The activation of EMT causes the cancer cells to migrate and invade. Decrease in epithelial markers, including E-cadherin and occludin, and an increase in the mesenchymal markers (N-cadherin, vimentin, and fibronectin) are considered as characteristics of EMT (31). A previous study (25) shows co-expression of COMP with mesenchymal markers and its negative correlation with the expression of epithelial markers. In the present study, we found that COMP was enriched in the EMT pathway in both BC and TC and correlated with some EMT markers. Therefore, COMP plays a role in the occurrence and progression of BC and TC through the EMT pathway.

Estrogen plays an important role in BC and TC. Estrogen acts via ER. In TC and BC, ERα enhances the migration and invasion of tumor cells by upregulating MMP-9 and downregulating E-cadherin (32–35). In addition (36, 37), estrogen can activate the MAPK pathway, the phosphatidylinositol 3-kinase (PI3K) signaling pathway, and MMPs by binding to membrane-associated estrogen receptor. Our analysis based on the SEER database found that HR-positive BC patients have higher risk of developing TC. Meanwhile, we found that COMP was up-regulated in ER-positive breast cancer. COMP was enriched in the estrogen response pathway in both BC and TC, and positively correlated with ESR1 expression. COMP may promote the development and progression of double primary cancer through the estrogen response pathway.

Based on the above findings, COMP is highly expressed in both TC and BC and has the same pathogenic pathway. Therefore, we hypothesized that COMP expression may also be elevated when patients developed BC or TC, thereby increasing the risk of another primary cancer through the same pathogenic pathway.

However, there are some limitations to this study. Compared with the general population, patients with BC or TC may undergo more examinations, thus, it is easier to find that they subsequently suffer from another cancer, which has a certain selection bias. However, how determining the true incidence of double primary cancer needs further designing of a more reasonable research program that includes appropriate research subjects. Such as prospective follow-up of healthy physical examination population. The function of COMP and the correlation of COMP with ER and EMT also need further experimental investigation. Moreover, the sodium iodide symporter in most DTC and BC are expressed and mediated by the radioactive iodine treatment. Therefore, the expression of sodium iodide symporter in double primary cancer and its relationship with COMP need to be further studied.

In conclusion, we found that the co-occurrence risk of BC and TC in the same individual is higher than in the general population. And double primary cancers share a common pathogenic mechanism. Overexpression of COMP could promote oncogenesis and progression of BC and TC patients through estrogen signaling and EMT pathways and may increase the risk of TC in BC patients or BC in TC patients.

## Data availability statement

The original contributions presented in the study are included in the article/**Supplementary Material**. Further inquiries can be directed to the corresponding authors.

## Ethics statement

All experimental procedures involving humans in this study were reviewed and approved by the Ethics Committee of China-Japan Union Hospital of Jilin University. Written informed consent was obtained from all patients.

## Author contributions

JF and MH performed statistical analysis and write the manuscript. QW performed bioinformatics analysis and did experiments. XZ collected clinical data and prepared some figures. XQ and KS collected part of the clinical data. XW participated in research conception, critical reading, and correction of the manuscript. GZ participated in research conception, supervised and coordinate research, and revised the manuscript. All authors contributed to the article and approved the submitted version.

## Supplementary material

Supplementary Figure 1
**(A)** Correlation between SLC5A5 expression and COMP mRNA expression in BC. **(B)** Correlation between SLC5A5 expression and COMP mRNA expression in TC. **(C)** Correlation between SLC5A5 expression and ESR1 mRNA expression in BC. **(D)** Correlation between SLC5A5 expression and ESR1 mRNA expression in TC. **(E)** Correlation between SLC5A5 expression and ESR2 mRNA expression in BC. **(F)** Correlation between SLC5A5 expression and ESR2 mRNA expression in TC.
