# Supplementary figures and images for: The clinical and genetic features in patients coexisting primary breast and thyroid cancers
Source: Front Endocrinol (Lausanne). 2023 May 9;14:1136120. doi: 10.3389/fendo.2023.1136120 (PMC10203615; doi:10.3389/fendo.2023.1136120)

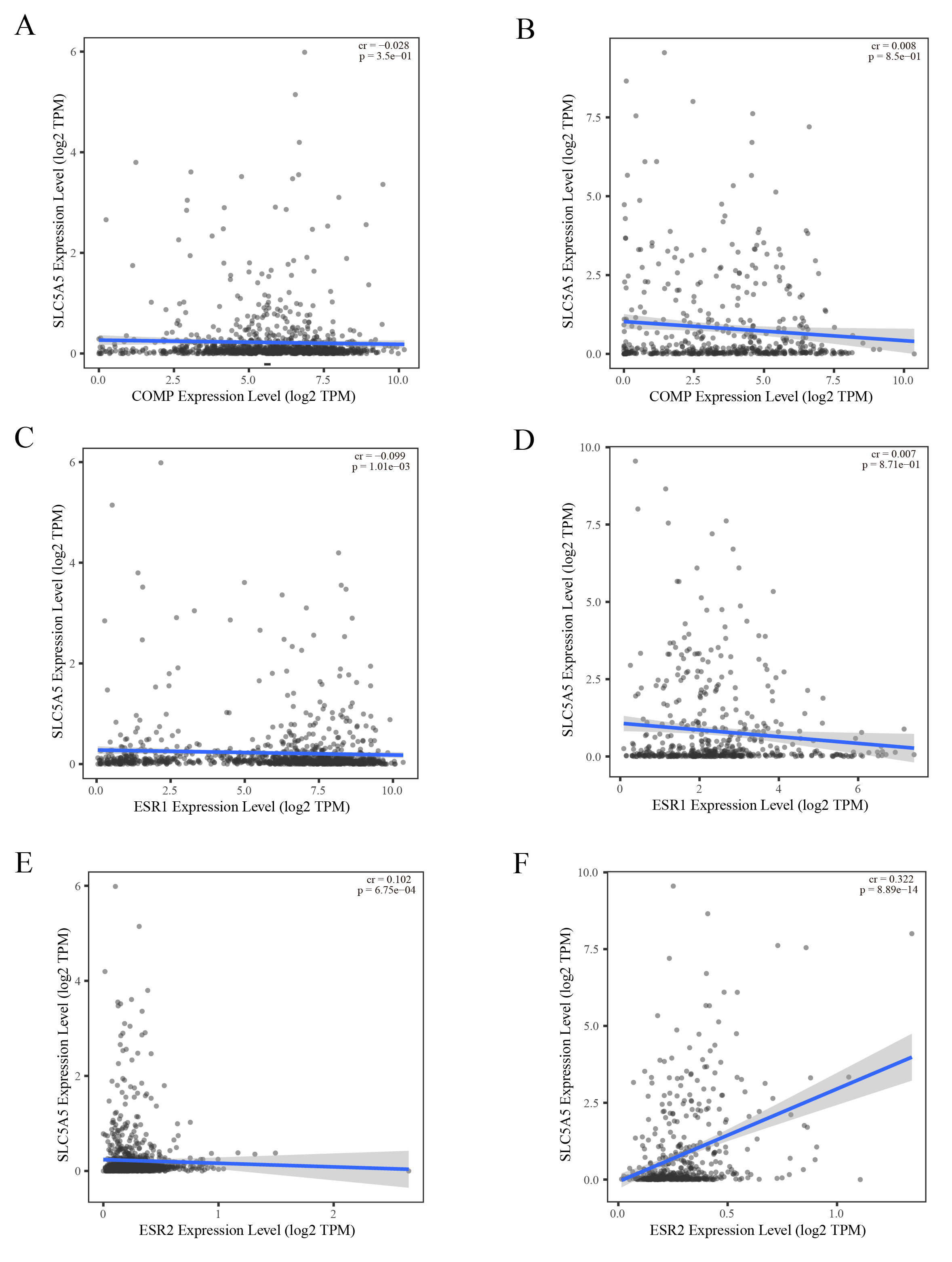

Supplement: Supplementary Figure 1 — (A) Correlation between SLC5A5 expression and COMP mRNA expression in BC. (B) Correlation between SLC5A5 expression and COMP mRNA expression in TC. (C) Correlation between SLC5A5 expression and ESR1 mRNA expression in BC. (D) Correlation between SLC5A5 expression and ESR1 mRNA expression in TC. (E) Correlation between SLC5A5 expression and ESR2 mRNA expression in BC. (F) Correlation between SLC5A5 expression and ESR2 mRNA expression in TC. [file Image_1.tif]
